# Profiles of PCSK9, SREBP-2, and histopathology in COVID-19 and non-COVID-19 critical illness

**DOI:** 10.1186/s12879-025-12129-1

**Published:** 2025-11-13

**Authors:** Florian Weber, Vlad Pavel, Martina Müller, Peter Boor, Lea Läber, Saskia von Stillfried, Christa Buechler

**Affiliations:** 1 Institute of Pathology, University of Regensburg, 93053 Regensburg, Germany; 2 Department of Internal Medicine I, Gastroenterology, Hepatology, Endocrinology, Rheumatology, Immunology, and Infectious diseases, Regensburg University Hospital, 93053 Regensburg, Germany; 3 Peter Boor, Saskia von Stillfried, Institute of Pathology, Medical Faculty, RWTH Aachen University, Aachen, Germany; 4 Department of Internal Medicine I, Regensburg University Hospital, Regensburg, Germany

**Keywords:** SARS-CoV-2, Sepsis, PCSK9, SREBP-2, Cholestasis

## Abstract

**Background:**

Severe illness caused by SARS-CoV-2 infection is associated with dysregulated cholesterol homeostasis. Proprotein convertase subtilisin/kexin type 9 (PCSK9), which regulates serum cholesterol levels, is induced in the plasma of patients with severe SARS-CoV-2 infection, compared to critically ill patients with other conditions. PCSK9 is primarily expressed in the liver, which is susceptible to damage during severe illness. Sterol regulatory element-binding protein 2 (SREBP-2) regulates PCSK9 expression, and higher activity of both PCSK9 and SREBP-2 is associated with liver injury and inflammation.

**Methods:**

Liver tissues from 20 COVID-19 and 20 pre-pandemic autopsy cases were analysed, matched for age, sex, and intensive care treatment. Hepatic PCSK9 and SREBP-2 protein levels were assessed via immunohistochemistry. Histological scores for steatosis, fibrosis, and cholestasis were recorded. Additionally, plasma SREBP-2 levels were measured by ELISA in 25 septic COVID-19 and 34 septic non-COVID-19 patients.

**Results:**

Across all cases, hepatocellular PCSK9 protein level was increased in the presence of cholestasis and positively correlated with hepatic SREBP-2 expression. No significant differences were observed between the COVID-19 and control groups regarding liver histology or hepatic PCSK9 and SREBP-2 protein levels. Plasma SREBP-2 levels were similar between COVID-19 and non-COVID-19 septic patients. Correlation analysis revealed positive associations between plasma SREBP-2, plasma PCSK9, and cholesteryl ester levels in the entire cohort, suggesting preserved SREBP-2 function during critical illness. Laboratory measures of liver disease in patients with and without SARS-CoV-2 infection were similar.

**Conclusion:**

Critically ill patients with and without SARS-CoV-2 infection exhibit comparable hepatic expression of PCSK9 and SREBP-2, as well as similar liver histology and comparable levels of aminotransferases, bilirubin, and gamma-glutamyl transferase, which suggests that SARS-CoV-2 does not directly cause liver injury. As our cohort was small, this suggestion needs to be confirmed by studying larger groups.

## Background

Bacterial and viral infections are common causes of sepsis, with severe acute respiratory syndrome coronavirus 2 (SARS-CoV-2) being a new source of sepsis and septic shock [1–3]. Severe inflammatory diseases are associated with a disturbance in lipid metabolism, and systemic cholesterol levels in these patients are reduced [4].

The physiological role of proprotein convertase subtilisin/kexin type 9 (PCSK9) is the regulation of serum cholesterol levels. By binding to the low-density lipoprotein receptor (LDL-R), PCSK9 inhibits its recycling, resulting in its degradation. Subsequently, cell surface levels of LDL-R and serum LDL clearance decrease, contributing to hypercholesterolemia [5]. It is notable that statins, which reduce serum cholesterol levels, have been shown to increase PCSK9 [5].

In inflammation and sepsis, serum PCSK9 levels are elevated compared to those of healthy controls [6–9]. Serum PCSK9 levels were higher in patients with moderate COVID-19 disease compared to controls and were further elevated in patients with severe disease [10]. SARS-CoV-2 infection was associated with higher plasma PCSK9 levels compared to similarly ill patients with sepsis/septic shock [8]. PCSK9 blockade during the inflammatory phase of SARS-CoV-2 infection reduced disease severity and mortality [11], indicating a role of high PCSK9 levels in COVID-19 severity.

PCSK9 is highly abundant in hepatocytes, and its expression is induced by sterol-regulatory element binding protein-2 (SREBP-2) [5, 9, 12]. Viral infection of cells in vitro has been shown to deplete cellular cholesterol levels, leading to activation of SREBP-2 [3, 13, 14]. Upon activation, SREBP-2 is processed by proteases and translocates from the cytoplasm to the nucleus [15]. SREBP-2 activation induced by proinflammatory cytokines of the SARS-CoV-2-infected peripheral blood mononuclear cells [16, 17] is associated with COVID-19 disease severity and may serve as a diagnostic tool and therapeutic target in these patients [16]. SREBP cleavage-activating protein and SREBP-2 are involved in the activation of the NLRP3 inflammasome, and SREBP-2 inhibition protected mice from inflammation [18].

SARS-CoV-2 primarily infects cells in the lungs [19]. However, not only are extrapulmonary clinical manifestations of SARS-CoV-2 recognised [20], but extrapulmonary evidence of cellular infection with the virus has also been demonstrated [21]. Molecular, clinical, and histopathological data support the idea that SARS-CoV-2 displays tropism towards the liver [22]. Chen et al. detected viral proteins in the hepatocytes of patients with severe COVID-19 disease but not in those with moderate COVID-19 [23]. One case report described viral particles in the hepatocytes of an immunosuppressed female patient [24]. Other studies could not provide evidence of SARS-CoV-2 liver tropism [25–27].

Liver abnormalities are commonly detected in patients with SARS-CoV-2 infection and range from mild elevations in aminotransferase levels to severe hepatic injury [22, 28–30]. The liver function abnormalities seen in critically ill patients with and without COVID-19 are largely similar, indicating that severe illness is the primary underlying factor [31].

Hepatitis C virus (HCV) infection induces hepatic and circulating PCSK9 levels [5]. HCV infection of hepatocytes also increased the expression of SREBP-2 and its target genes LDL-R and 3-hydroxy-3-methylglutaryl CoA reductase [32], suggesting that activation of SREBP-2 contributes to higher PCSK9 levels. SREBP-2 mRNA has also been found to be induced in the liver of patients with metabolic dysfunction-associated steatotic liver disease (MASLD) [33], and experimental studies suggest a role for this transcription factor in liver steatosis and fibrosis [15]. PCSK9 expression has been shown to increase with higher stages of fibrosis in the human liver [34]. However, data on the regulation of hepatic PCSK9 expression in chronic liver diseases are still inconclusive [5].

Circulating PCSK9 levels are elevated in patients with severe COVID-19, and its high expression in hepatocytes may indicate induction of PCSK9 synthesis in the liver [8, 10, 13]. SREBP-2 regulates PCSK9 and may also be activated in the liver of patients with severe COVID-19 disease [12, 14]. Moreover, the dysregulation of SREBP-2 and PCSK9 has been reported in cases of liver disease, such as hepatitis, suggesting the involvement of both proteins [35, 36].

In this study, PCSK9 and SREBP-2 protein levels in the liver of patients who died of SARS-CoV-2 infection during intensive care unit (ICU) treatment were compared with similarly ill ICU patients who died of other causes. Plasma SREBP-2 protein was also analysed in critically ill patients with and without SARS-CoV-2 infection.

## Materials and methods

### Case-control matching

*N* = 20 cases of COVID-19 patients who died in the ICU of RWTH Aachen University Hospital between March 2020 and April 2022 were matched 1:1 with *N* = 1020 control cases who died in an ICU of RWTH Aachen University Hospital between December 2007 and June 2022. One case with an undefined sex was excluded. Matching criteria were age difference ≤ 1 year and sex. A total of 20 cases were matched with 1020 controls, resulting in 20 matched controls (Table 1). Cases and controls were quality controlled microscopically for preservation of tissue morphology. One liver and one lung tissue sample per case were selected for further processing. Tissues of 14 male and six female patients of each cohort were used for analysis. The study protocol was approved by the ethical committee of the University Hospital Aachen (EK 304/20, EK 119/20, and EK 092/20) and was performed according to the updated guidelines of good clinical practice and the updated Declaration of Helsinki. Informed consent for autopsy was obtained from the legal representatives of the deceased patients.

### Plasma samples

The patients for analysis of plasma SREBP-2 were a randomly selected sub-cohort of patients described recently [8, 37]. These patients fulfilled sepsis criteria (Sepsis-3 [38]), 25 patients due to SARS-CoV-2 infection and 34 patients due to other causes. The Sepsis-related organ failure assessment (SOFA) scores of the patients were determined [39]. Plasma PCSK9 and cholesterol levels of the entire cohort were described previously [8, 37]. Patients with multi-resistant infections, viral hepatitis, or human immunodeficiency virus infection were excluded. The study protocol was approved by the ethical committee of the University Hospital of Regensburg (18-1029-101) and was performed in accordance with the updated guidelines of good clinical practice and the updated Declaration of Helsinki.

### Immunohistochemistry

The monoclonal PCSK9 antibody (order number: MA5-32843, Thermo Fisher Scientific; Waltham, MA, USA) and the monoclonal SREBP-2 antibody (order number: 6721-MSM2-P1, NeoBiotechnologies; Bath, UK) were diluted 1:200 (PCSK9) and 1:50 (SREBP-2), respectively. Staining protocols were established using different antibody dilutions and pre-treatment conditions on a control tissue microarray of normal human tissue (including skin, testis, colon, liver, placenta, spleen, kidney, and lung) until optimal staining was achieved, i.e., good staining intensity of the desired protein with minimal background staining.

For immunohistochemical analysis, 4–5 μm-thick sections of the liver tissue samples were subjected to deparaffinization, followed by antigen retrieval in Tris-EDTA buffer (pH 9) at 120 °C for 5 min. To inhibit endogenous peroxidase activity, a blocking solution from Dako (Glostrup, Denmark) was used. The sections were then incubated with the primary antibody for 30 min at room temperature. Staining was carried out using the Dako EnVision™+ Detection System, Peroxidase/DAB+, Rabbit (Dako, Glostrup, Denmark). The final step involved counterstaining the slides with hematoxylin.

The H-score was used for quantification, which is calculated as the percentage of positive cells multiplied by the staining intensity, ranging from 0 to 300. SREBP-2 is localized in the cytoplasm and the nucleus [40] and PCSK9 in the cytoplasm of the cell [5], and cytoplasmic SREBP-2 and PCSK9 were scored.

In lung tissues from matched cases and controls, SARS-CoV-2 nucleocapsid was detected by immunohistochemistry (Clone BSB-134, Bio SB, Santa Barbara, USA) according to the manufacturer’s protocol.

### Histological scores

The histological evaluation of the degree of liver fibrosis was graded using the Ishak fibrosis score, which is a seven-point grading system where 0 indicates no fibrosis and 6 indicates cirrhosis [41].

### SREBP-2 ELISA

The human SREBF2/SREBP-2 ELISA Kit (order number: LS-F12655) from LSBio (Newark, CA, USA) was used for quantitative protein analysis in plasma samples. The intra-assay CV < 5.6% and inter-assay CV < 8.3% were provided by the company. For analysis, plasma was diluted 1:5. All samples were measured in duplicate, and the mean values were used for calculations.

### Detection of the SARS-CoV-2 envelope gene

RNA isolation and reverse transcription polymerase chain reaction (RT-PCR) for the analysis of the SARS-CoV-2 envelope gene have been described previously [42].

### Statistics

Data in tables are given as median values and the range of the values. Boxplots were used to visualize the datasets in the figures. Figures 2b and 4b show the H-scores of the individual tissues. Statistical tests used were the Chi-square Test, Mann-Whitney U Test, and Spearman correlation (IBM Corp. Released 2019. IBM SPSS Statistics for Windows, Version 26.0. Armonk, NY: IBM Corp). A p-value < 0.05 was considered significant.

## Results

### SARS-CoV-2 detection in the lung

**Fig. 1:**
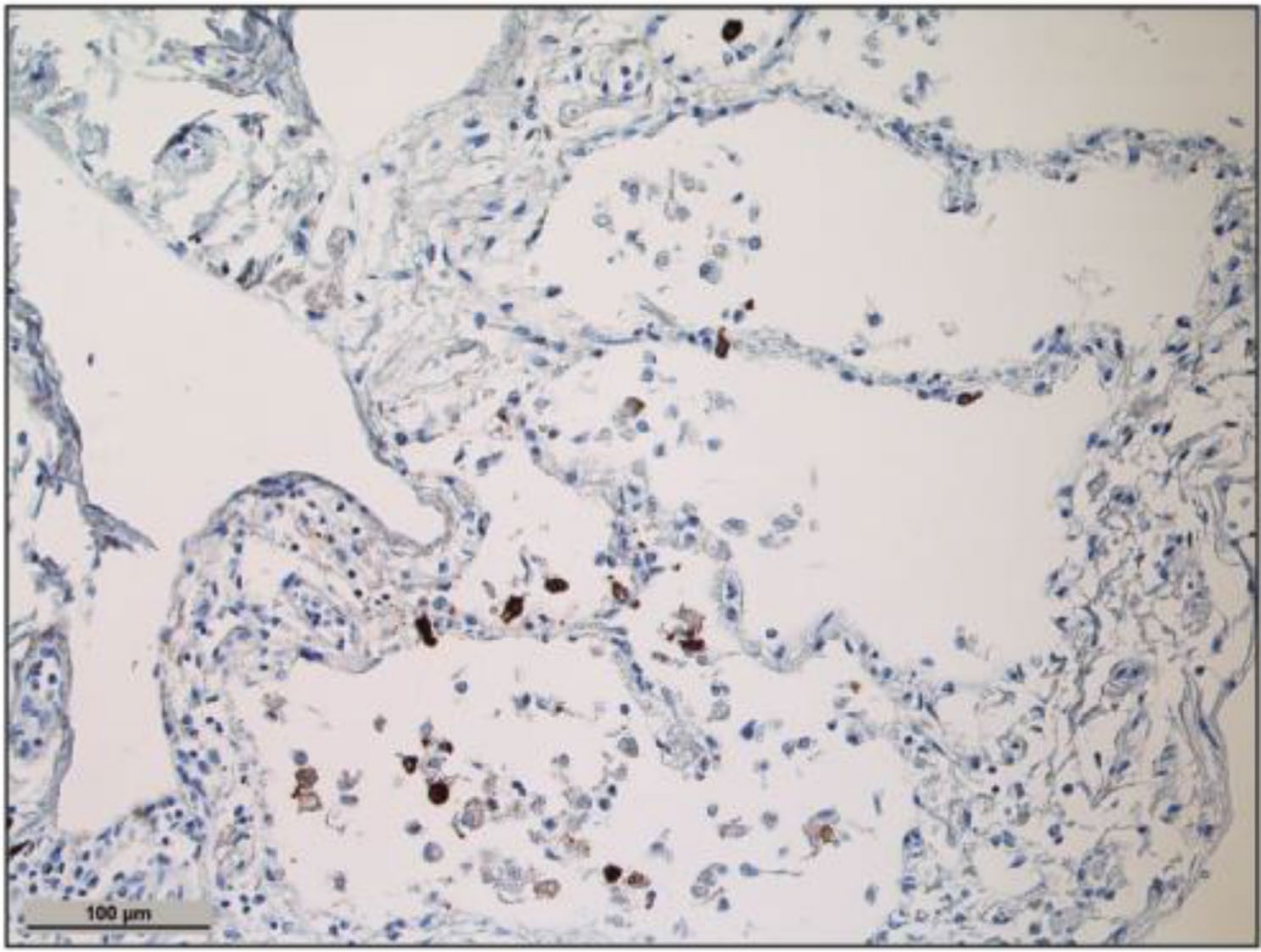
Detection of SARS-CoV-2 in the lung of a patient: intraalveolar cells (morphologically in line with alveolar macrophages) are highlighted in brown (SARS-CoV-2 nucleocapsid diaminobenzidin staining, hematoxylin counterstaining, scale bar = 100 μm)

SARS-CoV-2 nucleocapsid immunohistochemistry was positive in lung tissue in one of *n* = 20 COVID-19 autopsy cases (Fig. 1), but negative in the respective liver samples (data not shown).

Lung tissues of *n* = 18 out of *n* = 20 patients and liver tissue of *n* = 1 out of *n* = 6 patients with SARS-CoV-2 were positive for SARS-CoV-2 envelope (E) gene detected by RT-PCR (Table 1).

**Table 1:** Patient characteristics of SARS-CoV-2 autopsy cases and matched controls (E Gene = Envelope Gene). Samples with RT-PCR Ct (cycle threshold) values > 40 were considered negative [42]. N/A not tested

Case/control	Age range	Sex	Ct value SARS-CoV-2 E Gene from liver tissue	Ct value SARS-CoV-2 E Gene from lung tissue
Control	41–45	Male	N/A	N/A
COVID-19	41–45	Male	N/A	34.61
COVID-19	46–50	Male	N/A	33.47
Control	46–50	Male	N/A	N/A
COVID-19	46–50	Male	N/A	39.03
Control	46–50	Male	N/A	N/A
COVID-19	51–55	Female	N/A	21.42
Control	51–55	Female	N/A	N/A
COVID-19	56–60	Male	> 40	32.37
Control	56–60	Male	N/A	N/A
COVID-19	56–60	Male	> 40	29.15
Control	56–60	Male	N/A	N/A
COVID-19	56–60	Male	N/A	29.01
COVID-19	56–60	Male	N/A	36.01
Control	56–60	Male	N/A	N/A
Control	56–60	Male	N/A	N/A
COVID-19	56–60	Male	N/A	30.76
Control	56–60	Male	N/A	N/A
COVID-19	61–65	Female	> 40	39.01
Control	61–65	Female	N/A	N/A
COVID-19	61–65	Female	N/A	> 40
Control	61–65	Female	N/A	N/A
COVID-19	61–65	Male	N/A	32.61
Control	61–65	Male	N/A	N/A
COVID-19	66–70	Male	N/A	> 40
COVID-19	66–70	Male	> 40	31.98
Control	66–70	Male	N/A	N/A
Control	66–70	Male	N/A	N/A
Control	66–70	Female	N/A	N/A
COVID-19	66–70	Female	N/A	27.92
Control	66–70	Female	N/A	N/A
COVID-19	71–75	Female	33.11	27.23
COVID-19	71–75	Male	N/A	29.4
COVID-19	71–75	Male	N/A	26.09
Control	71–75	Male	N/A	N/A
Control	71–75	Male	N/A	N/A
COVID-19	71–75	Female	> 40	33.53
Control	71–75	Female	N/A	N/A
COVID-19	81–85	Male	> 40	24.92
Control	81–85	Male	N/A	N/A

Acute liver failure occurred in three controls and four cases (*p* = 0.531). Obesity and type 2 diabetes, which are risk factors for MASLD [43], were present in three controls and four cases (*p* = 0.597) and five controls and five cases (*p* = 0.596), respectively. Fatty liver was diagnosed in 3 cases (*p* = 0.115). Acute pancreatitis was found in 3 patients with SARS-CoV-2 infection and ulcerative colitis in 2 controls (*p* = 0.115 for both).

The duration of ICU stays differed between the two cohorts (*p* = 0.009), at 3 (1–109) days for controls and 16 (1–58) days for cases.

### Liver histology of cases and controls

Histologically defined steatosis grades (*p* = 0.142) and fibrosis stages (*p* = 0.611) of the two cohorts did not differ (Tables 2 and 3).

**Table 2:** Steatosis in the liver of deceased controls and COVID-19 patients

Steatosis %	0	5	10	20	30	40	60	70
Controls	8	7	1	2	0	1	0	1
Cases	10	1	3	3	2	0	1	0

**Table 3:** Fibrosis stages in the liver of deceased controls and COVID-19 patients

Scoring (Ishak)	0	1	2	3	4	5	6
Controls	10	4	1	1	1	1	2
Cases	10	7	0	2	0	0	1

The fibrosis stages are concordant to the clinical data where 3 controls and 1 case were diagnosed as having cirrhosis (*p* = 0.363).

Eight of the 20 controls (40%) and seven of the 20 COVID-19 patients had cholestasis (35%), with a similar prevalence in both cohorts (*p* = 0.500).

Sex-specific analysis was done in the entire cohort, and steatosis, fibrosis and cholestasis prevalence of both sexes were comparable (*p* = 0.779, *p* = 0.823 and *p* = 0.291, respectively).

### PCSK9 protein in the liver

**Fig. 2:**
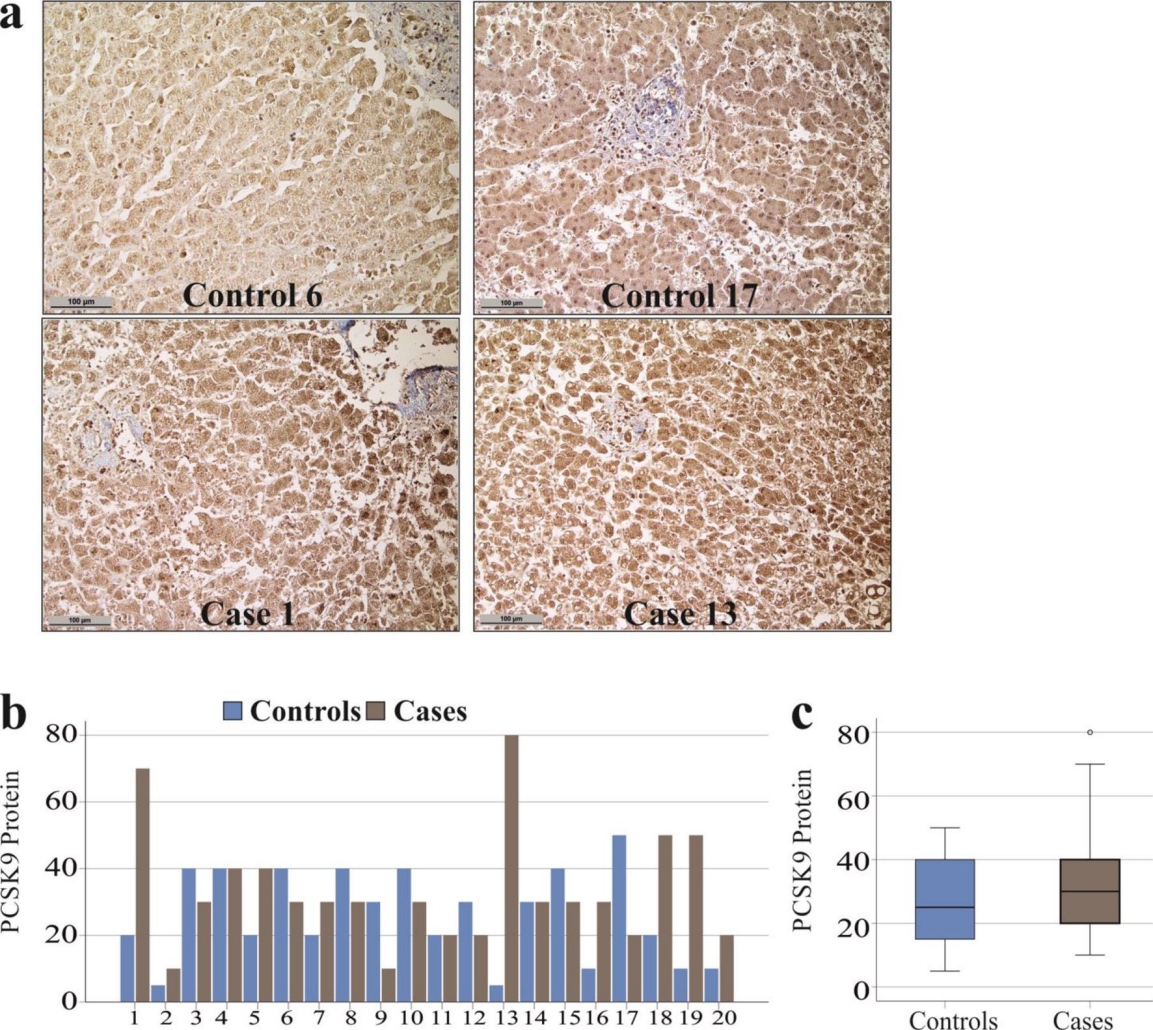
Detection of PCSK9 protein in the liver of controls and patients with COVID-19. (**a**) Immunohistochemistry of PCSK9 in the liver of two controls (control 6: H-score = 40, control 17: H-score = 50 for) and two patients with COVID-19 (case 1: H-score = 70, case 13: H-score = 80) (scale bar = 100 μm); (**b**) Individual H-scores for PCSK9 protein levels in the liver of *n* = 20 controls and *n* = 20 patients with COVID-19; (**c**) PCSK9 protein levels (H-scores) of 20 controls and 20 cases

PCSK9 protein was detected by immunohistochemistry (Fig. 2a) and the protein levels of hepatocytes were scored. PCSK9 protein was higher in *n* = 9 cases compared to controls, similar in *n* = 3 cases compared to controls and lower in *n* = 8 cases compared to controls (Fig. 2b). The expression of PCSK9 in hepatocytes from patients with SARS-CoV-2 infection relative to matched controls was similar (*p* = 0.763) and ranged from 33% to 1600% relativ to the respective controls.

**Fig. 3:**
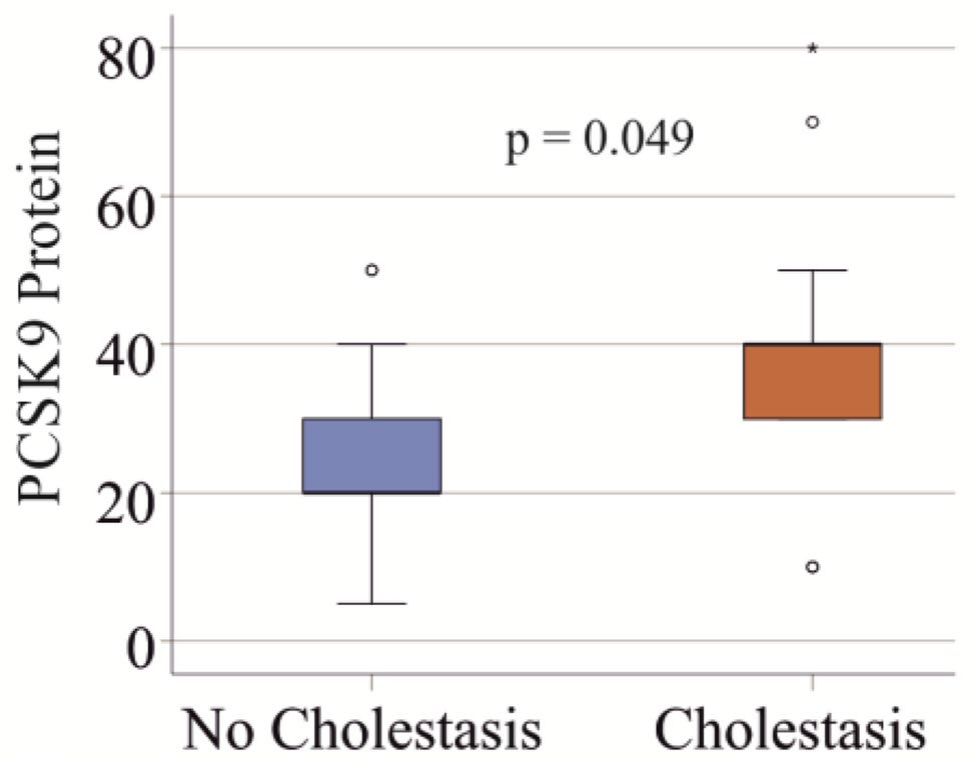
PCSK9 protein levels determined by immunohistochemistry in the liver of patients with and without cholestasis. The H-scores of PCSK9 protein levels, quantified by immunohistochemistry, are shown for the 25 patients without cholestasis and the 15 patients with cholestasis

PCSK9 protein levels in hepatocytes did not correlate with the degree of steatosis or fibrosis in the entire cohort (*p* > 0.05 for both). However, in the entire cohort, patients with cholestasis had higher levels of hepatocyte PCSK9 (*p* = 0.049) (Fig. 3).

The 12 females and 28 males had similar PCSK9 protein in the liver (*p* = 0.567).

### SREBP-2 protein in the liver

**Fig. 4:**
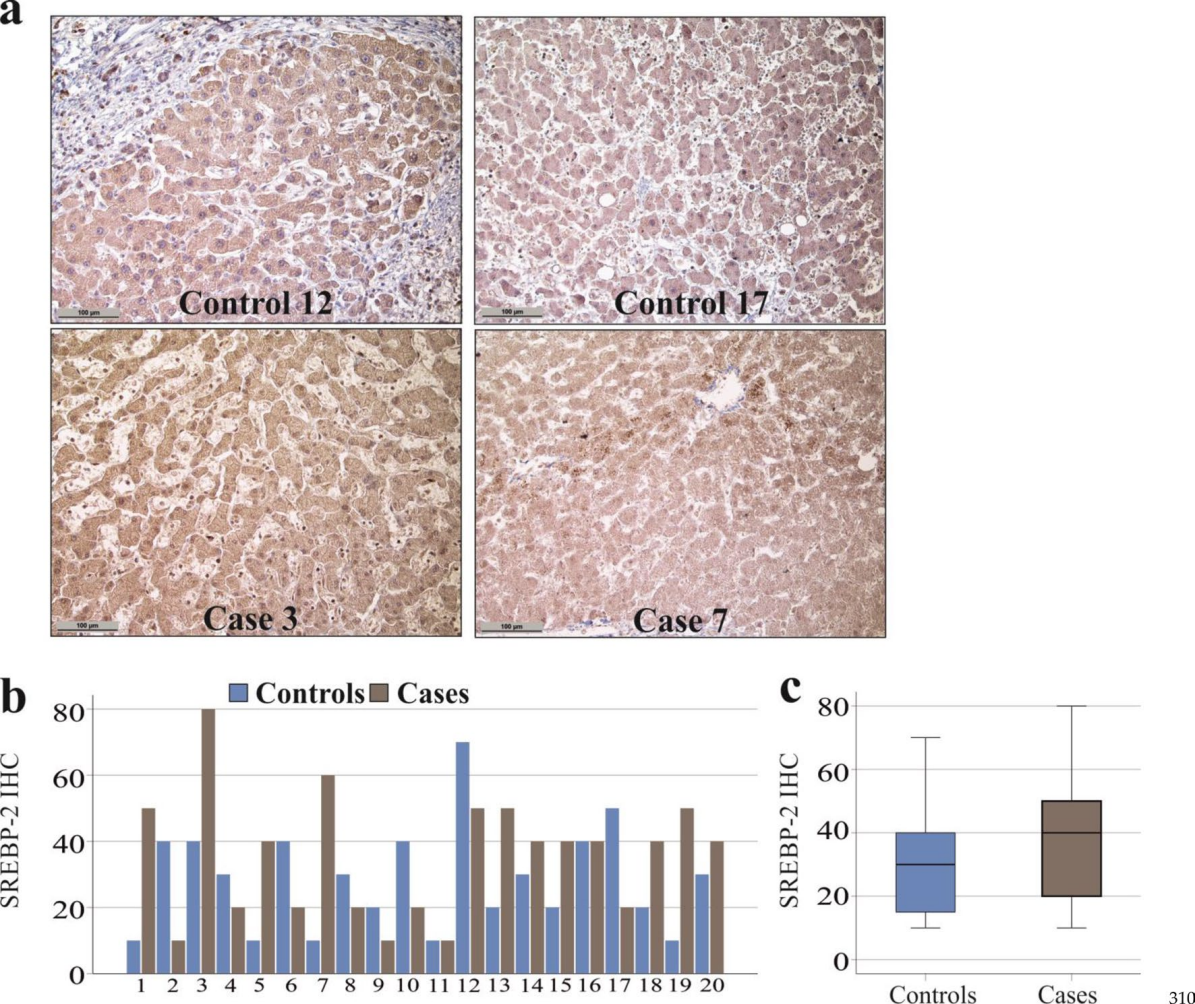
Detection of SREBP-2 protein in the liver of controls and patients with COVID-19. (**a**) Immunohistochemistry of SREBP-2 in the liver of two controls (control 12: H-score = 70, control 17: H-score = 50) and two patients (case 3: H-score = 80 and case 7: H-score = 60) with COVID-19 (scale bar = 100 μm); (**b**) Individual H-scores of SREBP-2 protein in the liver of *n* = 20 controls and *n* = 20 patients with COVID-19; (**c**) SREBP-2 protein levels (H-scores) of 20 controls and 20 cases

SREBP-2 regulates PCSK9 expression [12] and was also quantified in the liver of patients with COVID-19 and matched controls (Fig. 4a). In the liver tissue of *n* = 10 COVID-19 patients SREBP-2 was higher, in *n* = 2 tissues the expression was similar, and was lower in tissues of *n* = 8 patients compared to controls (Fig. 4b). The expression of SREBP-2 in hepatocytes from COVID-19 patients was 117% (25% − 600%) relative to the levels in the control livers, and was similar to the matched controls (*p* = 0.602).

In the entire cohort, SREBP-2 protein staining results did not correlate with the degree of steatosis or fibrosis. Cholestasis was not related to higher SREBP-2 protein staining (*p* > 0.05 for all). PCSK9 and SREBP-2 protein levels were correlated with each other (*r* = 0.511, *p* = 0.001). Females and males had similar SREBP-2 protein in the liver (*p* = 0.619).

### Statin use of cases and controls

Statin use was documented for 4 controls and 8 cases, and was higher in the latter cohort (*p* = 0.029). Prior medication of 5 controls and 9 cases was not known. In the entire cohort, statin users had similar SREBP-2 (*p* = 0.349) and PSCK9 (*p* = 0.649) levels as non-users. Prevalence of cirrhosis, acute liver failure, obesity, diabetes, steatosis, fibrosis and cholestasis and days in ICU of patients with and without statins did not differ (*p* > 0.05).

### SREBP-2 protein in the plasma of severely ill patients with and without SARS-CoV-2 infection

Plasma SREBP-2 protein levels were measured by ELISA in septic patients with and without SARS-CoV-2 infection. This cohort is a sub-cohort of patients with systemic inflammatory response syndrome (SIRS) or sepsis, as recently described [8, 37]. At the time of this study, sufficient plasma was available from 25 patients with confirmed SARS-CoV-2 infection, and all of these patients were included. All patients with SARS-CoV-2 infection had sepsis, and only non-SARS-CoV-2 patients with sepsis were included, meaning that 37 patients with SIRS were excluded. Of the patients with SARS-CoV-2, two had liver cirrhosis. Of the non-infected patients, one with cirrhosis was included, meaning that a further 22 patients with liver cirrhosis were excluded. Of the remaining 72 patients, those were selected for whom sufficient plasma (50 µl per patient) was available to determine SREBP-2.

Plasma PCSK9 protein levels, determined by ELISA, and the cholesterol levels of the entire cohort have been described previously [8, 37]. The cohorts for analysing SREBP-2 protein levels by ELISA are described in Table 4.

**Table 4:** Characteristics of critically ill patients with and without SARS-CoV-2 infection. IL-6 levels were determined in 22 COVID-19 and 28 non-COVID-19 patients. Data are given as the median (minimum–maximum) * *p* < 0.05

Parameters	SARS-CoV-2 infected	Not SARS-CoV-2 infected
Males/Females	17/8*	26/8*
Age (years)	63 (29–80)	54 (29–83)
BMI (kg/m^2^)	28 (21–45)	26 (18–55)
C-reactive protein mg/L	129 (27–472)	201 (40–503)
Procalcitonin ng/mL	0.6 (0.1–65.4)*	2.9 (0.1–112.3)*
SOFA Score	10 (5–16)*	7 (2–18)*
IL-6 pg/mL	47 (6–1810)	98 (7–3817)
Leukocytes *n* × 10^9^/L	8.8 (2.8–18.5)	11.3 (0.3–40.4)
Neutrophils n/nL	6.5 (0.1–48.4)	9.5 (0–70.2)
Basophils n/nL	0.03 (0–0.60)*	0.05 (0–0.90)*
Eosinophils n/nL	0.04 (0–8.80)	0.04 (0–1.75)
Monocytes n/nL	0.62 (0–10.90)	0.81 (0.08–45.00)
Lymphocytes n/nL	0.68 (0.08 − 28.60)	1.02 (0.29–16.80)
Immature granulocytes n/nL	0.24 (0–3.84)	0.27 (0–6.19)
PCSK9 ng/mL	341 (14–858)*	240 (40–822)*
Cholesterol nmol/mL	2829 (988–5727)	2360 (1476–4423)
Cholesteryl Ester nmol/mL	1784 (255–3668)*	1168 (138–2746)*
Bilirubin mg/dl	0.6 (0.2–7.3)	1.0 (0.2–18.6)
Albumin g/L	28 (20–37)*	25 (17–34)*
Aspartate Aminotransferase	54 (21–126)	54 (11–635)
Alanine Aminotransferase	36 (11–283)	35 (9–227)
Gamma-glutamyl transferase	198 (22–1266)	142 (27–479)*
Dialysis	11**	6**
Ventilation	25***	12***
Vasopressor therapy	23***	16***
Non-survival	9**	2**
Pneumonia	24***	2***
Pancreatitis/Urosepsis/Cholangitis	0/0/0***	12/4/3***
Liver cirrhosis	2	1
Statin Yes/No/n.d.	3/22/0	1/32/1

Patients with COVID-19 had lower procalcitonin levels and fewer basophils (Table 4). There were more female patients in this cohort. Patients with SARS-CoV-2 infection had a higher SOFA score than patients with sepsis who were not infected with SARS-CoV-2 (see Table 4). Furthermore, more patients with SARS-CoV-2 infection were on dialysis, ventilation, and vasopressor therapy. Mortality of patients with COVID-19 was higher.

Non-surviving patients with SARS-CoV-2 infection had a higher SOFA score (*p* = 0.006), whereas PCSK9, SREBP-2, and cholesteryl ester levels were similar between survivors and non-survivors (*p* > 0.05 for all). Almost all of the patients with SARS-CoV-2 infection had pneumonia and did not have other underlying conditions, such as pancreatitis.

Very few patients were taking statins (Table 4). Those with SARS-CoV-2 infection who were on statin therapy had similar levels of PCSK9, total cholesterol, and SREBP-2 to those with SARS-CoV-2 infection who were not taking statins (*p* = 0.235, 0.269 and 0.361, respectively).

**Fig. 5:**
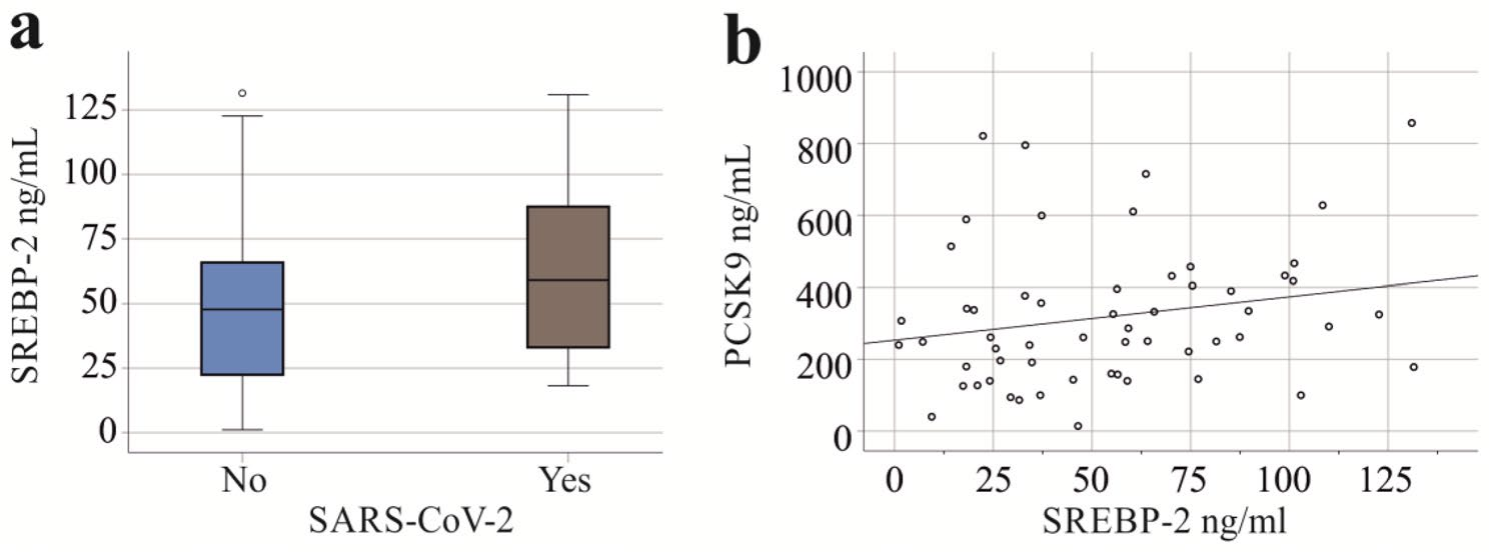
SREBP-2 in the plasma of severely ill patients without and with SARS-CoV-2 infection. (**a**) SREBP-2 in the plasma of severely ill patients without (No) and with (Yes) SARS-CoV-2 infection; (**b**) Correlation of SREBP-2 and PCSK9 in the plasma of all patients

Plasma PCSK9 levels and cholesteryl ester levels of patients with SARS-CoV-2 infection were higher compared to those of patients not infected with this virus, as has been described before [8] (Table 1). Laboratory parameters of liver disease (bilirubin, aminotransferases, and gamma-glutamyl transferase) of patients with and without SARS-CoV-2 infection did not differ (Table 4). Albumin of patients with COVID-19 was higher compared to severely ill patients not infected by this virus (Table 4). Plasma SREBP-2 protein levels in septic patients with and without SARS-CoV-2 infection were not found to be significantly different between groups (*p* = 0.174; Fig. 5a).

Since plasma SREBP-2 levels were similar across the patient cohorts, correlation analysis was performed on the entire cohort. SREBP-2 in plasma did not correlate with the SOFA score (*r* = − 0.211, *p* = 0.119), total plasma cholesterol (*r* = 0.259, *p* = 0.067), CRP (*r* = 0.140, *p* = 0.290), age (*r* = -0.016, *p* = 0.893), or BMI (*r* = 0.104, *p* = 0.427). The positive correlation of SREBP-2 with PCSK9 (*r* = 0.249, *p* = 0.062, Fig. 5b) was almost significant. Cholesteryl ester levels were positively (*r* = 0.317, *p* = 0.023) and procalcitonin levels (*r* = -0.335, *p* = 0.011), as well as IL-6 (*r* = -0.299, *p* = 0.017), were negatively correlated with plasma SREBP-2 levels.

## Discussion

This study assumes that SREBP-2 function remains intact in cases of severe illness due to a positive correlation between SREBP-2 and PCSK9 plasma levels, as well as cholesteryl ester levels. Furthermore, it is suggested that it is severe illness rather than SARS-CoV-2 infection itself that is associated with hepatic dysfunction. This is based on the comparable expression of PCSK9 and SREBP2 protein levels in the hepatocytes from severely ill patients with and without SARS-CoV-2 infection, as well as similar liver histology and levels of systemic markers of liver disease in cases and controls.

Previously, our group reported higher levels of PCSK9 in the plasma of SARS-CoV-2 infected sepsis patients compared to patients with different disease etiologies [8]. Here, we analysed whether elevated circulating PCSK9 levels in patients with severe SARS-CoV-2 infection can be attributed to the induction of PCSK9 synthesis and its regulation by SREBP-2 in the liver [5, 8]. Of note, there was no difference in the expression of either protein between the cases and the controls. The levels of PCSK9 and SREBP-2 proteins were positively correlated in the liver, which is consistent with these proteins being closely associated [5].

Levels of PCSK9 in the blood of patients with sepsis are increased [6, 8, 44], but its association with disease severity remains unclear [6, 8, 10, 44]. Patients with severe SARS-CoV-2 infection had comparable levels of C-reactive protein (CRP), interleukin-6 (IL-6), and most types of leukocytes compared to sepsis patients with different disease etiologies. In line with previous studies, the procalcitonin levels of patients with SARS-CoV-2 infection were lower [45, 46]. Patients with viral infections had an elevated SOFA score, and a greater proportion of patients in this cohort did not survive, indicating that they were more severely ill. Notably, SARS-CoV-2 infection was associated with higher levels of cholesteryl esters and a tendency towards higher serum cholesterol levels, consistent with increased PCSK9 [5]. Cholesterol levels were also higher in the serum of patients with severe SARS-CoV-2 infection than in patients in the intensive care unit with cardiogenic shock, suggesting that this might be a general characteristic of these patients [47].

The normal expression of PCSK9 protein in hepatocytes, alongside higher plasma PCSK9 levels, suggests reduced PCSK9 elimination. The LDL receptor on hepatocytes plays a crucial role in removing lipopolysaccharide (LPS) from the circulation [48]. LPS levels in patients with septic shock who were infected with SARS-CoV-2 were higher than in septic shock patients who were not infected with this virus [49]. Therefore, the higher levels of PCSK9, cholesterol [37, 47], and LPS [49] in SARS-CoV-2 infection suggest that hepatic LDL-R expression is reduced in these patients. However, the effect of severe SARS-CoV-2 on hepatic LDL-R expression has not been analysed, and further studies are needed to identify these underlying mechanisms.

Although the origin of plasma SREBP-2 and the specific molecular regions detected by the ELISA remain unclear, plasma SREBP-2 levels showed a positive correlation with both PCSK9 and cholesteryl ester concentrations. This is largely consistent with the activities of SREBP-2, the major transcription factor for cholesterol biosynthesis [15], which also increases PCSK9 expression in hepatocytes [12]. However, plasma SREBP-2 protein levels were similar in patients with and without COVID-19, and thus may not contribute to higher plasma PCSK9.

SREBP-2 protein levels and processing are increased during cholesterol depletion [15, 50]. Levels of the SREBP-2 C-terminal fragment, which is released from this transcription factor during proteolytic activation, were increased in the blood of patients with severe COVID-19 compared to patients with less severe disease and healthy controls [16]. However, it is unclear whether the C-terminal SREBP-2 fragment is specific for severe COVID-19 disease or is a feature of critical illness of different etiologies [16].

In particular, proteolysis of SREBP-2 promotes activation of the inflammasome and production of inflammatory proteins such as tumor necrosis factor [16, 17]. Plasma SREBP-2 protein levels analysed in our study negatively correlated with procalcitonin and IL-6, showing associations with anti-inflammatory rather than proinflammatory pathways. Analysing the SREBP-2 C-terminal fragment in patients with sepsis caused by different factors may provide further insight into the role of this transcription factor in inflammation [16].

Statins, which reduce plasma cholesterol levels and increase PCSK9 [51], are commonly used drugs in Germany [52]. Higher levels of PCSK9 were observed in sepsis patients prescribed statins compared to those with sepsis who did not receive statins [6]. Total cholesterol levels in sepsis patients who take statins are either higher or similar to those of non-users [53, 54]. Studies have demonstrated that statins may reduce the mortality of patients with sepsis independently of their cholesterol-lowering activities [13, 55, 56]. In the present cohort analysed for plasma SREBP-2, only four patients were prescribed statins. This low number may have precluded the identification of significant effects of this therapy. In the cohort where liver tissue was obtained, statin use was documented for four controls and eight cases, with a higher prevalence in the latter group. In the entire cohort, statin users had similar SREBP-2 and PCSK9 levels to non-users. The prevalence of cirrhosis, acute liver failure, obesity, diabetes, steatosis, fibrosis, cholestasis, and the number of days patients spent in the ICU did not differ between those who took statins and those who did not. However, our cohorts were small, so this study is not suitable for drawing final conclusions about the effect of statins on hepatic PCSK9 or SREBP-2, or on the prevalence of metabolic and liver diseases.

SARS-CoV-2 was not detected by immunohistochemistry in the liver of our patients who died from severe COVID-19 disease, which is consistent with other studies [25, 26]. The virus was detected in the lung tissue of one patient out of 20 by immunohistochemistry. Using RT-PCR analyses of the E gene, *n* = 18 out of *n* = 20 SARS-CoV-2 autopsy lung tissues and *n* = 1 out of *n* = 6 SARS-CoV-2 autopsy liver tissues were positive. This shows that detecting SARS-CoV-2 in the liver is rare. A previous study found viral proteins in the livers of three patients who died from severe disease symptoms, but not in the livers of three patients with moderate symptoms [23]. SARS-CoV-2 RNA was detected in the livers of patients who died with COVID-19 in 13/20 cases by PCR and in 9/20 cases by in situ hybridization. Spike protein was found in the liver of four out of 20 cases, and nucleocapsid protein in 15 out of 20 cases. This study showed that the virus infected endothelial cells, Kupffer cells and portal macrophages, but not hepatocytes [27]. The detection of SARS-CoV-2 RNA and proteins in the liver does not definitively prove infection, as viral particles may be derived from the circulation [57]. The frequency of liver tropism of SARS-CoV-2 and the types of cells infected are currently unclear.

Cholestasis was diagnosed in up to 40% of patients with sepsis [58] and in 40 to 50% of critically ill COVID-19 patients [59, 60], showing a similar prevalence in both conditions. In our COVID-19 patients, 35% had cholestasis, which is comparable to the 40% of controls with cholestasis. Patients with cholesterol gallstones exhibited higher PCSK9 expression in their livers compared to those without gallstones [61]. PCSK9 protein levels were also increased in our septic patients with cholestasis, though this effect was marginal.

Similar prevalence and extent of liver steatosis and fibrosis were observed in cases and controls, suggesting that liver dysfunction is not specific to viral infection. Liver steatosis (defined as > 5% fat in the liver) was found in five controls and nine patients with COVID-19, with a similar prevalence. This is not very different from the prevalence of liver steatosis in the German population, where almost 38% have liver steatosis [62]. Advanced fibrosis was found in three controls and one COVID-19 patient, representing 15% and 5% of patients, respectively. Advanced fibrosis occurs in approximately 1% of the general population, with a higher prevalence in patients with severe disease [63], in accordance with our observation. Levels of laboratory markers for liver disease did not differ significantly between cases and controls, which is consistent with comparable liver function in both groups. Patients with SARS-CoV-2 had higher albumin levels, indicating a better liver synthesis rate.

It should be noted that the SOFA score of the patients with confirmed cases of SARS-CoV-2 in our cohort was higher, indicating more severe illness. Besides the fact that cases had a longer ICU stay than controls, whose relevance to the current study is unclear, there were no differences between cases and controls with respect to obesity or type 2 diabetes. The percentage of patients with acute liver failure was similar in both cohorts.

PCSK9 expression has been shown to increase with the extent of fibrosis in the human liver [34], and SREBP-2 contributes to fibrogenesis [33, 40]. However, hepatocyte PCSK9 and SREBP-2 protein expression was not correlated with the stage of steatosis and fibrosis in severe illness. To what extent steatosis and fibrosis develop and progress during critical illness remains to be addressed by future longitudinal studies.

This study has limitations. The cohorts were small, and SREBP-2 activity was not measured in the liver and plasma. The commercial SREBP-2 assays used in this study did not specify which regions of the protein were detected. Liver cholesterol levels were also not determined. In addition, plasma from deceased patients was unavailable. Liver histology was not documented before severe illness, as liver biopsies were not available at that time. Some autopsy cases had limited clinical data available, and information about parenteral nutrition was provided for very few cases. This information was not included in the current study.

## Conclusion

In summary, this study revealed a positive association between SREBP-2 and PCSK9 plasma levels, as well as cholesteryl ester levels, in patients with sepsis. This suggests that SREBP-2 function is preserved. The comparable levels of PCSK9 and SREBP-2 proteins in hepatocytes from cases and controls suggest that the elevated plasma PCSK9 and cholesteryl ester levels observed in patients with SARS-CoV-2 infection are unlikely to result from changes in hepatic synthesis. This analysis found no evidence that SARS-CoV-2 infection exacerbates liver steatosis, fibrosis, or cholestasis in patients with severe illness. However, given the limited size of the cohort, these findings require corroboration in larger cohorts.

## Data Availability

Data can be obtained from the authors on request. Immunostaining of all tissues can be requested from Florian Weber, and ELISA data from Christa Buechler. All other data are included in the manuscript.
